# Supplementary material for: Gestational Factors throughout Fetal Neurodevelopment: The Serotonin Link
Source: Int J Mol Sci. 2020 Aug 14;21(16):5850. doi: 10.3390/ijms21165850 (PMC7461571; doi:10.3390/ijms21165850)
Supplement: Supplementary file 1 [file ijms-21-05850-s001.pdf]

Table S1. Characteristics of human literature reviewed

Specifics	Time window	Effects on fetal 5-HT system	Effects on offspring's brain/body	Effects on offspring's behaviour	Study design	Data source	Case definition	Exclusion criteria	Comparison group	Reference
**3.1. 5-HT-ergic Maternal Genotype Influences the 5-HT System, Neurodevelopment, and Behaviour in the Offspring**										
Maternal TPH1 loss-of-function mutations	Prenatal	N.D.	N.D.	1.5 to 2.5 times higher ADHD scores	Case-control study	National registry of adult ADHD patients in Norway & National Public Registry	A clinical diagnosis of ADHD corresponding to DSM-IV criteria, ≥18 years old, males & females	N.D.	Paternal TPH1 loss-of-function mutations & healthy controls	Halmoy et al. (2010)
Maternal 5-HTTLPR-L allele	Prenatal	N.D.	N.D.	Higher risk for ASD	Cohort study	Autism Genome Research Exchange, Stanford, Tufts & Vanderbilt	Classification of Autism on the Autism Diagnostic Interview-Revised, ±8 years old, males & females	Additional children from family of whom 1 male & female offspring were already included	Offspring genotype & Parent-of-origin	Kistner-Grifin et al. (2011)
Maternal depression with or without depressed children	Prenatal	N.D.	*Depressed mother and child:* decreased CpG DNA methylation at repeat AluJb element in *SLC6A4* promoter region	NS difference in depression between 5-HTTLPR variations	Case-control study	Basic Health Unit of Uberaba, Minas Gerais, Brazil	Children, males & females, 6-12 years old	Mothers with psychiatric disorders other than depression, mothers receiving psychological treatment; children with history of organic diseases or sensory deficiencies	Healthy mothers with healthy children	Mendonca et al. (2019)
Lower levels of maternal whole-blood 5-HT levels	Prenatal but measured ±9 years after birth	N.D.	N.D.	Most severely affected ASD phenotypes	Case-control study	University of Illinois at Chicago & University of Texas Southwestern Medical Center	A diagnosis of Autistic Disorder, Asperger's Disorder or Pervasive Developmental Disorder Not Otherwise *(Specified based on DSM-IV-TR criteria, confirmed with both the Autism Diagnostic*	Participants whom themselves or at least one of his/her parents take medications acting on 5-HT system	Father & child levels of whole-blood 5-HT levels	Montgomery et al. (2018)

							Observation Schedule & the Autism Diagnostic Interview-Revised), ±9 years old, males & females			
5-HTTLPR-SL children from SS mothers	Prenatal	N.D.	Increased somatosensory cortex grey matter density	Increased performance in visuomotor performance	Cohort study	Generation R Study	5-HTTLPR- SL children from SS mothers, ±7 years old, males & females	N.D.	5-HTTLPR-SL children from LL mothers	Van der Knaap et al. (2014)

3.2 Maternal 5-HT-ergic Related Diets Influence the Tryptophan Pathway, Neurodevelopment, and Behaviour in the Offspring

Maternal alcohol consumption	First trimester	Decreased 5-HT levels in maternal serum (measured at GW9-11)	N.D.	N.D.	Cohort study	Kuopio University Hospital	A total score of ≥ 8 on the Alcohol Use Disorders Identification Test and/or alcohol use during pregnancy	N.D.	Non-smoking, healthy mothers with appropriate for gestational age infants from a non-complicated vaginal birth & normal outcome	Lehikoinen et al. (2018)
Maternal alcohol consumption	Prenatal	N.D.	Decrease in brain volume; decrease in 5-HT in medial prefrontal cortex, increase in striatal dopamine transporter binding	*All subjects:* ADHD but only correlation with dopamine	Case-control study	Kuopio University Hospital	Children with fetal alcohol effects or fetal alcohol syndrome, 5-16 years old.	N.D.	Children with normal MRI and no fetal alcohol syndrome; but with other clinical diagnoses	Riikonen et al. (2005)

3.3. Maternal Stress Affects the 5-HT System, Neurodevelopment, and Behaviour in the Offspring

High maternal anxiety *(> 38 on Trait Anxiety Scale)*, mostly comorbid with depression & anger	Prenatal and postnatal	N.D.	Decrease in 5-HT & dopamine *(measured in urine)*; Increase in relative activation of right frontal EEG	Affect sleep states; decrease performance on the Brazelton Neonatal Behavior Assessment Scale	Case-control study	Hospital prenatal clinics	Infants, males & females	N.D.	Low maternal anxiety *(< 38 on Trait Anxiety Scale)*	Field et al. (2003)

Maternal antidepressan, mainly SSRIs intake or untreated maternal depression *(HAM-D ≥ 8)*	Prenatal *(Minimum of 75 days during third trimester)*	N.D.	*Maternal depression:* increased connectivity between hubs in left anterior cingulate, insula, caudate & amygdala. *SSRI intake:* increase hub value in right medial frontal orbital gyrus & Heschl's gyrus compared to depression-only group	N.D.	Cohort study	University of British Columbia Research	Infants, males & females. *(MRI at postnatal day 6, temperament measurement at 6 months)*	Substance abuse, bipolar disorder & significant medical or obstetrical or fetal conditions	Non-depressed, SSRI-unexposed group	Rotem-Kohavi et al (2019)

3.4. Maternal Intake of 5-HT-ergic Medication Alters 5-HT Levels in the Offspring and Affects their Neurodevelopment and Behaviour

3.4.1. Maternal Intake of 5-HT Receptor (Ant)Agonist Might Affect the Unborn Child

Maternal Triptan intake *($5\text{-}HT_{1B/1D}$ receptor agonist)*	First trimester *(GW0-12)* or second & third trimester *(GW 13-birth)* or use during pregnancy with unknown timing	N.D.	N.D.	*First trimester intake:* increased risk for attention problems	Cohort study	Norwegian Mother and Child Cohort Study & Medical Birth Registry of Norway	Z ≥ 1.5/T≥65 on the Child Behavior Checklist, a validated, parent-reported measure, 3-year-old	Infants not born alive, born with major congenital malformations or chromosomal abnormalities, women who reported triptan exposure but did not report whether exposure occurred prior to or during pregnancy	Healthy mothers or mothers with migraines but without Triptan use or mothers who used Triptans prior to pregnancy only	Wood et al. (2016)

3.4.2. Maternal SSRI Intake is Associated with Neural Changes and Behaviour in the Offspring in Humans

Case-control & cohort studies

Maternal depression *(lifetime Axis*	*Depression/ anxiety:* Lifetime or	N.D.	*Lifetime depression:* associated with baseline & mean	N.D.	Cohort study	Emory Women's Mental Health Program & a	Infants, males & females, ±6 months old	A lifetime diagnosis of schizophrenia or bipolar disorder,	Women with no lifetime history of Axis I mood	Brennan et al. (2008)

I diagnoses of major depression, dysthymia or both) or comorbid with anxiety disorder; with & without psychotrophic medication *(mainly SSRIs)*	perinatal or prenatal or neonatal *Medication*: prenatal		cortisol concentrations *Perinatal depression*: associated with cortisol reactivity *Perinatal depression comorbid anxiety:* associated with cortisol reactivity *Medication:* decrease reactivity & moderate relation between maternal disorder and fetal cortisol			research subject pool maintained by the Emory University Psychology Department		or a primary lifetime diagnosis of an anxiety disorder but no lifetime diagnosis of depression/ dysthymia	or anxiety disorders	
Maternal SSRI intake	Prenatal	N.D.	N.D.	NS increased risk for ASD after adjustment for treatment probability or after comparison to unexposed siblings	Cohort study	Health administrative data from Ontario, Canada	Children, males & females, 4-10 years old (mean 4.95)	Mothers who filled only a single SSRI prescription during pregnancy, children who did not survive after 2 years of age	SSRI-unexposed group	Brown et al. (2017b)
Maternal SSRI intake *(paroxetine, fluoxetine, sertraline, venlafaxine, citalopram, escitalopram)*	Prenatal	N.D.	Decrease in serum total reelin protein levels (females)	NS neonatal behaviour including motor development, cry quality & sleep	Cohort study	Reproductive Mental Health Clinic	Infants, males & females	N.D.	No psychiatric medications	Brummelte et al. (2013)
Maternal SSRI intake or untreated maternal depression	Prenatal	N.D.	N.D.	*SSRI exposure & maternal depression:* Increase in externalizing behaviours *SSRI exposure vs control:* Increase in internalizing behaviours	Cohort study	Norwegian Mother and Child Cohort Study	Children, males & females, 5-6 years old	Multiple births; *Control group:* analgesic use	Healthy controls	Hermansen et al. (2016)

Maternal SSRI intake *(self-reported receiving: fluoxetine, escitalopram, citalopram, sertraline, venlafaxine)*	Prenatal	N.D.	Increase in grey matter volume in amygdala & insula, increase white matter structural connectivity between these regions (independent on dosage used)	N.D.	Cohort study	Columbia University Medical Center and New York State Psychiatric Institute	Infants, males & females, ±3,5 weeks old	N.D.	Untreated prenatal maternal depression *(score ≥ 16 at Center for Epidemiological Studies depression scale)* or healthy controls	Lugo-Candelas et al. (2018)
Maternal SSRI intake	Prenatal: early (week 0-16) or mid (week 17-28) or late (week 29+)	N.D.	N.D.	*SSRI exposure, late pregnancy:* increase risk for anxious/depressive behaviours by age 5	Cohort study	Norwegian Mother and Child Cohort Study & Medical Birth Registry of Norway	Children, males & females, 1.5-5 years old	Unknown timing of antidepressant exposure or multiple pregnancies	Untreated prenatal maternal depression or anxiety	Lupattelli et al. (2018)
Maternal SSRI intake *(prescription: fluoxetine, citalopram, paroxetine, sertraline, fluvoxamine, escitalopram)*	Prenatal *(SSRI purchase during 30 days before pregnancy – delivery)*	N.D.	N.D.	Increase risk of depression; NS ASD & ADHD	Cohort study	Finland Medical Birth Register, Register of Congenital Malformations, Hospital Discharge Register, Drug Reimbursement Register, Population Register	Depression disorders & unspecified affective disorders *(ICD-10 F32-39)*; ASD *(F84, but excluding Rett's syndrome, F84.2)*; ADHD *(F90)*,14.9 years old	A depression diagnosis only during the first 2 years of life	Unexposed mothers *(without psychiatric disorders & SSRI intake)* or Psychiatric mothers without SSRI intake or SSRI discontinued mothers whom purchased during 1 year up to 3 months before pregnancy	Malm et al. (2016)
Maternal SSRI intake	Prenatal	N.D.	Decrease in neonatal S100B serum levels; increase in maternal S100B serum levels	N.D.	Cohort Study	University of British Columbia Research & Children's and Women's Health Centre of British Colombia Research	Infants, males & females, at delivery and first 48 hours of life	Other psychotropic or antidepressant use during pregnancy	SSRI-unexposed group	Paluwski et al. (2009)

Maternal SSRI intake	Prenatal	N.D.	Increased serum CBG levels which predicted a smaller diurnal change in salivary cortisol *(controlling for maternal depression)*	N.D.	Cohort study	University of British Columbia Research & Children's and Women's Health Centre of British Colombia Research	Infants, males & females, at delivery & 3 months of age	N.D.	SSRI-unexposed group	Pawluski et al. (2012a)
Maternal SSRI intake	Pre-gestation and/or prenatal	N.D.	Alterations in microstructural & metabolic development of the basal ganglia, thalamus & occipital cortex	NS cognitive, language & motor outcomes (18 months of age)	Cohort study	British Columbia Women's Hospital	Preterm-born neonates (24-32 gestational week), males & females,	N.D.	SSRI-unexposed group	Podrebarac et al. (2017)
Maternal antidepressant intake (mainly SSRIs)	Prenatal *(first trimester)*	N.D.	N.D.	NS increase in ASD, ADHD	Cohort study	Swedish Multigeneration Register, Prescribed Drug Register, National Patient Register, National Crime Register, Swedish Register Of Education	Males & females	Diagnoses before 2 years of age, multiple births	Antidepressant unexposed group	Sujan et al. (2017)
Systematic reviews & meta-analyses										
Maternal SSRI intake	Prenatal	N.D.	N.D.	Increased ASD diagnosis	Systematic review & meta-analysis	4 cohort & 3 case-control studies	N.D.	Studies reporting outcomes in a non-uniform exposure time window or non-extractable estimates of SSRI exposure / ASD offspring	N.D.	Andalib et al. (2017)
Maternal SSRI intake	Prenatal *(first trimester or any use during pregnancy)*	N.D.	N.D.	*Case-control studies, first trimester intake:* Increase in ASD *(after adjustment for maternal mental illness)*	Systematic review & meta-analysis	2 cohort & 4 case-control studies	N.D.	Studies including non-SSRI antidepressants; studies including ASD-like symptoms without diagnosis of ASD. One case-control and one cohort study were excluded due to overlap	SSRI-unexposed group	Brown et al. (2017a)

								between studies & low-quality assessment score		
Maternal SSRI intake	Prenatal	N.D.	N.D.	Possible increase in ASD (6/8 studies) or in ADHD (1/2 studies not reporting an increase in ASD)	Systematic review	4 cohort & 4 case-control studies	N.D.	SSRI use for treatment of ASD symptoms, articles describing non-primary data	N.D.	Gentile (2015)
Maternal SSRI intake	Pre-gestation or first trimester or second trimester or third trimester	N.D.	N.D.	*All except third trimester:* increase risk of ASD	Systematic review & meta-analysis	4 cohort & 5 case-control studies	N.D.	A case-control study was excluded due to overlap between studies & low-quality assessment score	N.D.	Kaplan et al. (2016)
Maternal SSRI intake or SSRI-discontinuation until 3 months before pregnancy or untreated maternal depression	Prenatal	N.D.	N.D.	*SSRI intake & untreated maternal depression:* increase risk of ASD and thus confounding by indication	Systematic review & meta-analysis	4 cohort studies	N.D.	Case-control studies; One cohort study was excluded for not including a point estimate consistent with inclusion criteria	SSRI-unexposed group	Kaplan et al. (2017)
Maternal SSRI intake	Prenatal	N.D.	N.D.	Increase risk of ASD but not increased when compared to non-SSRI depressed group	Systematic review & meta-analysis	5 cohort & 6 case-control studies	N.D.	N.D.	SSRI-unexposed group *(healthy & disease women without SSRI-exposure)*	Kobayashi et al. (2016)
Maternal SSRI intake	Prenatal	N.D.	N.D.	Increase risk of ASD	Systematic review & meta-analysis	2 cohort & 5 case-control studies	N.D.	N.D.	N.D.	Man et al. (2015)
Maternal SSRI intake	Pre-gestation and/or prenatal	N.D.	N.D.	Increase risk of ADHD can be partially explained by confounding by indication	Systematic review & meta-analysis	5 cohort & 3 case-control studies *(including 3 sibling matched studies that*	N.D.	N.D.	N.D.	Man et al. (2018)

						compared the exposure & outcome status among siblings born to the same mother)				
Maternal SSRI intake	Pre-conception and/or first and/or second and/or third trimester	N.D.	N.D.	*Case-control studies:* increase risk for ASD (whole pregnancy or any trimester) *Cohort studies:* increased risk for ASD (preconception exposure only)	Systematic review & meta-analysis	3 cohort & 7 case-control studies	N.D.	Studies lacking a control group	SSRI-unexposed or non-ASD groups	Mezzacappa et al. (2017)
**3.5. Maternal immune activation affects the tryptophan pathway and neurodevelopment of offspring**										
Maternal bacterial infection or placentae incubated in bacterial endotoxin lipopolysaccharide	Prenatal	Alter placental enzyme expression<br><br>*endotoxin:* increase blood levels and placental output of kynurenine and quinolinic acid	N.D.	N.D.	Form of case-control study	Monash Medical Center	Placentae and cord blood from preterm deliveries with or without bacterial infection; placentae cultured in lipopolysaccharide	N.D.	Placentae and cord blood from term deliveries	Manuelpillai et al. (2004)

5-HT: serotonin; 5-HTTLPR: 5-HT transporter-linked polymorphic region; ADHD: attention deficit hyperactivity disorder; ASD: autism spectrum disorders; CBG: corticosteroid binding globulin; GW: gestational week; ICD: International Classification of Disease; N.D.: not determined; NS: not significant; P: postnatal day; SSRI: selective serotonin reuptake inhibitor; TPH: tryptophan hydroxylase.

Table S2. Characteristics of animal literature reviewed

Specifics	Time window	Effect on offspring's 5-HT synthesis	Effects on offspring's brain development	Effects on offspring's behaviour	Species	Strain	Sex offspring	Control definition	Reference
**3.1. 5-HT-ergic Maternal Genotype Influences the 5-HT System, Neurodevelopment, and Behaviour in the Offspring**									
TPH1 HET embryo's from KO dam	Prenatal	N.D.	Altered shape & reduced mitotic activity in the roof of the neopallial cortex and altered shape hindbrain regions	N.D.	Mouse	C57BL/6	N.D.	TPH1 KO embryo's from TPH1 HET dam	Cote et al. (2007)
5-HT$_{1A}$ receptor KO & WT offspring from HET dam	Prenatal	N.D.	Delayed development of the ventral dentate gyrus (adulthood)	Increased anxiety-like behaviour (adulthood)	Mouse	Swiss Webster	N.D.	5- HT$_{1A}$ WT offspring from WT dam	Gleason et al. (2010)
TPH1 KO embryo's from TPH1 KO dam	Prenatal	N.D.	*TPH1-KO embryos:* decreased cell proliferation *(E18)*	N.D.	Mouse	C57BL/6J	-	TPH1 WT embryo's from TPH1 WT dam	Hadden et al. (2017)
SERT Ala56 KI embryo's from KI dam	Prenatal	Decreased forebrain 5-HT levels (E14.5)	Broadening of 5-HT-sensitive somatosensory TCAs (E14.5 & E18.5)	N.D.	Mouse	129S6/S4	N.D.	SERT Ala56 WT embryo's from WT dam	Muller et al. (2017)
5-HT$_{1A}$ receptor-KO offspring from HET or KO parents; WT offspring from HET parents	Prenatal	N.D.	N.D.	*Offspring from KO and HET mothers:* reduced ultrasonic vocalization (between P4 & P12; offspring genotype affects P4 only)	Mouse	Swiss-Webster	Males & females	WT offspring from WT parents	Van Velzen & Toth (2010)
**3.2 Maternal 5-HT-ergic Related Diets Influence the Tryptophan Pathway, Neurodevelopment, and Behaviour in the Offspring**									

**3.2.1. Maternal Tryptophan-Related Diets Affect the Placental Tryptophan Pathway in Animals**									
TRP-restricted diets									
TRP-injection through stomach intubation (0.05, 0.2, 0.5 or 1 g TRP/kg)	Prenatal *(E17)*	Dose-related increase in of TRP in the placenta; dose-related increase of TRP in the brain for at least 24 hours; increase of 5-HT in the brain for at least 1 hour; *TRP doses > 0.2 g:* increase of 5-HIAA in the brain for at least 24 hours. (E17)	N.D.	N.D.	Rat	Sprague Dawley	N.D.	Saline solution	Arevalo et al. (1991)
TRP-free diet	Prenetal *(E5-P0)*	NS whole-brain 5-HT (P0)	Reduction 5-HT synthesizing neurons, disturbed neuronal migration and altered dorsal raphe topography at DRN (P0)	N.D.	Rat	Wistar	Males	Standard chow & TRP-free diet with TRP supplements	Flores-Cruz & Escobar (2012)
TRP deprived diet: tortilla diet	Neonatal *(P1-28)*	N.D.	Reduced dendritic spine density & dendrite arbor atrophy in hippocampal pyramidal neurons; abnormal dendrite swelling and reduced hippocampal proliferation (adolescent)	N.D.	Rat	Wistar	Males	Tortilla diet supplemented with 0.2% L-TRP	Zhang et al. (2006)
TRP-free diet	Neonatal *(P0-8)*	Decreased PFC 5-HT levels; Decreased 5-HT turnover rate in hippocampus; increased 5-HT turnover rate in striatum (adulthood)	Decrease in striatal BDNF levels; NS hypothalamic BDNF levels (adulthood)	Increased anhedonia-like behaviour (adulthood)	Mouse	CD-1	Males	Standard diet: 1.4 g TRP/kg	Zoratto et al. (2013)

TRP-enhanced diets									
TRP enriched diet (10 g TRP/kg)	Perinatal *(E1-PW20)*	Increased peripheral 5-HT levels & increased peripheral TPH activity (PW 1 up to 20)	N.D.	N.D.	Rat	Sprague Dawley	Males & females combined	Standard chow: 2.2 g TRP/kg	Castrogi-ovanni et al. (2014)
TRP enriched diet: 13.5 g TRP/kg	Pre-gestational & perinatal *(2 weeks before mating-weaning)*	Decreased brainstem & frontal cortex 5-HT levels & TPH activity (P0 up to P10: brainstem; to P30: frontal cortex)	Reduction of 5-HT accumulation in frontal cortex synaptosomes (until P30)	N.D.	Rat	Wistar	Males	Standard chow: 3.5 g TRP/kg	Huether et al. (1992)
TRP enriched diet: 10 g TRP/kg	Perinatal *(E1-P20)*	Increased peripheral 5-HT levels (juvenile)	N.D.	N.D.	Rat	Sprague Dawley	N.D.	Standard chow: 2.2 g TRP/kg	Musumeci et al. (2014)
TRP enriched diet: 50 g TRP/kg	Pre-gestational & prenatal *(10 days before mating-E17.5)*	Increased peripheral non-proteinaceous L-TRP (E12.5 & E17.5)	N.D.	N.D.	Mouse	ICR	N.D.	20% Casein diet & 20% Casein diet with 20 g TRP/kg	Tsuji et al. (2013)
Protein and carbohydrate diets									
Protein restricted diet: isocaloric 6% casein + L-methionine	Pre-gestational & prenatal *(female diet: 5 weeks before until the end of pregnancy; male diet: 1 week prior to mating)*	NS 5-HT hippocampal tissue concentration; Decreased 5-HT uptake sites	Decreased 5-HT fibre density in dentate gyrus & hippocampus and 5-HT_{1A} receptor in hippocampus	N.D.	Rat	Sprague Dawley	N.D.	Isocaloric 25% casein diet + L-methionine	Blatt et al. (1994)
Protein restricted diet: 6% casein	Pre-gestational & prenatal *(female diet: 5 weeks before until the end of pregnancy; male diet: 1 week prior to mating)*	NS 5-HT hippocampal tissue concentration; Increased 5-HT and 5-HIAA efflux from hippocampus (P220)	NS differences in maximal 5-HTT binding or 5-HTT affinity (P220)	N.D.	Rat	Sprague Dawley	Males	25% casein diet	Chen et al. (1992)
Protein restricted diet: isocaloric 80 g protein/kg	Perinatal *(E1-P21)*	Decreased forebrain & hindbrain 5-HT levels (E13 & E17); Increased adult 5-HT	N.D.	N.D.	Rat	Wistar	*Embryo's:* N.D. *Adult:* males & females	170 g protein/kg	Honorio de Melo Martimia-no et al. (2017)

		levels in the brainstem, hippocampus & cerebral cortex							
Protein restricted diet: 6% casein	Pre-gestational & prenatal *(female diet: 5 weeks before until the end of pregnancy; male diet: 1 week prior to mating)*	Increased hypothalamus & hippocampus 5-HT; increased hypothalamus 5-HIAA (juvenile *(P9)*)	Decreased hypothalamus volume, decreased CORT response to isolation stress (juvenile *(P9)*)	N.D.	Rat	Sprague Dawley	Males & females combined	25% casein diet	Kehoe et al. (2001)
Isocaloric carbohydrate restricted diet (0%, 4%, 12% fructose or glucose)	Prenatal *(E0-E21;On E21 fetuses were delivered by caesarean section)*	*0%-12%:* dose-dependent increase in 5-HT and 5-HIAA. decrease in both at 0%; increase in both at 12% (E21)	N.D.	N.D.	Rat	Sprague Dawley	N.D.	Isocaloric control diet: 60% fructose or glucose	Koski et al. (1993)
Protein restricted diet: isocaloric 80 g protein/L	Prenatal *(E1-P0)*	Increased fetal whole-brain 5-HT levels (E16.4) & hypothalamus (P0); NS hypothalamic 5-HT levels (juvenile & adulthood)	Decreased fetal whole-brain 5-HT_{2C} receptor protein; only decreased in hypothalamus (P0 up to adulthood)	N.D.	Rat	Wistar	*Embryo's:* males & females; *Pups:* males	200 g protein/L	Martin-Gronert et al. (2016)
Protein restricted diet: 6% protein	Perinatal *(female diet: 5 weeks before pregnancy P21)*	Increased base 5-HT release in dorsal hippocampus, increased 5-HT suppression following electrical stimulation of RN	N.D.	N.D.	Rat	Sprague Dawley	N.D.	25% protein diet with microdialysis of DRN	Mokler et al. (1999)
Protein restricted diet: isocaloric 60 g protein/L	Pre-gestational & perinatal *(5 weeks before pregnancy – P0)*	Increased ventral medial prefrontal cortex 5-HT levels (adulthood)	N.D.	N.D.	Rat	Long Evans	Males	250 g protein/L	Mokler et al. (2019)
Protein restricted diet: isocaloric 6% casein	Pre-gestational & prenatal *(female diet: 5 weeks before pregnancy until the end of lactation)*	Increased 5-HT, 5-HIAA & TRP in telencephalon, diencephalon, midbrain, pons-medulla and cerebellum (juvenile/adolescent)	Increased free plasma TRP (juvenile/adolescent)	N.D.	Rat	Sprague Dawley	Males & females	25% casein diet	Resnick & Morgane (1984)

Protein restricted diet: 80 g protein /kg + 1.3 g TRP/kg	Prenatal *(E2-20)*	NS 5-HT levels fetal plasma & liver (E20)	N.D.	N.D.	Rat	Wistar	N.D.	180 g protein/kg	Sano et al. (2016)
Protein restricted diet: 8% casein supplemented with 0.4% methionine	Pre-gestational & prenatal *(5 weeks before E0 to P0, P5, P11, P16, P21, P30, P60, P145 or P300)*	*At birth:* increased 5-HIAA *All ages:* increased 5-HIAA and 5-HT levels, mainly in diencephalon, midbrain & pons-medulla	N.D.	N.D.	Rat	N.D.	Males & females	25% casein diet or standard chow	Stern et al. (1974)
Protein reduced diet: isocaloric 100 g protein/L	Prenatal *(E1-P0)*	NS hippocampal & PFC TRP & 5-HT levels (juvenile)	Reduction hippocampal 5-HT_{1A} receptor function (females juvenile & adulthood)	Increased sensitivity to stress (adult females) but NS anxiety-like behaviour	Rat	Wistar	Males & females	Control diet: 200 g protein/L	Ye et al. (2018)

3.2.2. Maternal High Fat Diet Reduces 5-HT Production in the Animal Offspring

High-fat diet: 60% energy from fat	Pre-gestational & perinatal (3 weeks before E0 until 3 weeks of lactation)	N.D.	Increase BDNF mRNA in the dorsal hippocampus and 5-HT_{1A} & $GABA_{Aa2}$ mRNA in the ventral hippocampus. NS 5-HT_{1B} mRNA in both regions (adulthood)	Increased anxiety-like behaviour, NS conditioned fear response & exploratory behaviour (adulthood)	Mouse	C57BL/6N	Males & females	Normal laboratory chow	Peleg-Raibstein et al. (2012)
High-fat diet: 32% calories from fat	Pre-gestational & prenatal & entire life	Increased mRNA TPH2 rostral DRN; NS caudal DRN (E130); Decreased CSF 5-HT levels (1 year old)	Increased mRNA 5-HT_{1A} receptor in DRN; Unaffected hypothalamic 5-HTT expression & 5-HT immunoreactivity (1 year old)	Increased anxiety-like behaviour (females; 1 year old)	Macaca Fuscata	-	Males & females	Control diet: 13% calories from fat	Sullivan et al. (2010)
High-fat diet: 35.7% calories from fat	Prenatal or Perinatal (until weaning)	*Both time periods:* Reduction in TPH2 mRNA expression in MRN & DRN (juvenile)	*Perinatal:* Reduction in 5-HT positive fibres in the mPFC & increased plasma cortisol levels (juvenile)	*Both time periods:* increased anxiety-like behaviour (juvenile)	Macaca Fuscata	-	Males & females combined	Control diet: 11.9% calories from fat	Thompson et al. (2017)

3.2.3. Maternal Alcohol Consumption Reduces Fetal 5-HT Production

Alcohol liquid diet: 36% ethanol-derived calories (*ad libitum*) or 18% ethanol-derived calories (isocaloric-pair-fed to a 36% animal)	Prenatal *(E1-E20)*	*High ethanol females:* decreased striatum 5-HT & striatum 5-HIAA (juvenile)	N.D.	N.D.	Rat	Sprague Dawley	Males & females	Liquid diet: 0% ethanol-derived calories (isocaloric-pair-fed to a 36% animal)	Clausing et al. (1996)
In vitro neuronal cell culture from rhombencephalic tissue *(E14)*, treated with 0-100 mM ethanol	Last 24 hours *in vitro* (day 5-6) or last 4 days in vitro (day 2-6)	N.D.	Decrease in 5-HT neurons; increased apoptosis	N.D.	Rat	Sprague Dawley	-	*In vitro* neuronal cell culture from rhombencephalic tissue *(E14)*, treated with 0-100 mM ethanol	Druse et al. (2004)
In vitro neuronal cell culture from rhombencephalic tissue *(E14)*, treated with 50 mM ethanol & 5-HT$_{1A}$ agonist: 100 nM ipsapirone	*Ethanol:* last 24 hours *in vitro* (day 5-6) + duration of ipsapirone treatment *Ipsapirone:* last 4 or 6 hours *in vitro* (day 6)	N.D.	*Ethanol:* reduced expression of pro-survival genes: XIAP, cIAP1, cIAP2, Bcl-2, and Bcl-xl *Ethanol+ipsapirone:* partial restoration of pro-survival gene expression: XIAP and Bcl-xl; increased 5-HT$_{1A}$ receptor expression (adulthood)	N.D.	Rat	Sprague Dawley	-	*In vitro* neuronal cell culture from rhombencephalic tissue *(E14)* treated with 100 nM ipsapirone	Druse et al. (2006)
Alcohol liquid diet: isocaloric 2.5 to 5% (w/v) ethanol + 5-HT$_{2A/2C}$ agonist: DOI 1 mg/kg	Prenatal *(Alcohol: E10-E20 & agonist: E13-E19)*	*Alcohol:* decreased 5-HT levels fetal whole-brain	*Alcohol:* 5-HT-ergic neurons in MRN & DRN & decreased brain growth *Agonist:* reverse effects on 5-HT-ergic neurons (E19/20)	N.D.	Rat	Sprague Dawley	Males	Combination of standard chow & saline injection	Ishiguro et al. (2016)
Alcohol injections: 500 or 1000 or 2000 mg/kg/day	Prenatal *(E15-P0)*	Dose-dependent decrease of 5-HT synthesis & TPH expression in DRN (3 & 5 weeks old)	N.D.	N.D.	Rat	Sprague Dawley	Males & females combined	Saline injection (subcutaneously)	Kim et al. (2005)
Alcohol: 5.1 g/kg/day by gavage	Prenatal *(E1-E20 or E20 only)*	*E1-E20 & E20:* decreased 5-HT & 5-HIAA (young adolescent)	*E1-E20:* increased GABA levels (young adolescent)	N.D.	Rat	Sprague Dawley	N.D.	Standard chow; pair-fed/intubated or ad libitum	Maier et al. (1996)

Alcohol liquid diet: E10-E12: 2.5% (w/v) E13-E15: 4.0% (w/v) E16-E21: 5.0% (w/v)	Prenatal *(E10-E21)*	N.D.	Decreased number of TPH-ir cells in paradorsal RN and MRN (adult)	Reduced activity in novel cage; increased amount of open arm entries in elevated plus maze; increased freezing behaviour (adult)	Rat	Sprague Dawley	Males	Ad libitum control diet (standard chow) or pair-fed liquid control diet (isocaloric, alcohol substituted for sucrose)	Ohta et al. (2010)
Alcohol liquid diet: 4.49% (v/v); 25% ethanol-derived calories	Prenatal *(E7-E18)*	N.D.	*Ethanol vs chow:* decrease in posterior medial barrel subfield area, average individual barrel area and B-row barrel volume; decreased layer IV barrel count; decreased neuron count	N.D.	Mouse	C57BL/6	N.D.	Ad libitum control diet (standard chow) or pair-fed liquid control diet (isocaloric, alcohol substituted for maltose dextrin)	Powrozek & Zhou (2005)
Alcohol liquid diet: 4.49% (v/v); 25% ethanol-derived calories	Prenatal *(E7-E13)*	Reduced levels of 5-HT (E13)	Reduced levels of GABA, NS glutamate (E13)	N.D.	Mouse	C57/BL6	N.D.	Pair-fed liquid control diet (isocaloric, alcohol substituted for maltose dextrin)	Sari et al. (2010)
Alcohol liquid diet: 4.49% (v/v); 25% ethanol-derived calories	Prenatal *(E7-E18)*	N.D.	Reduced 5-HT-ergic neurons in MRN & DRN (E18)	N.D.	Mouse	C57BL/6	N.D.	Isocaloric-pair-fed liquid control & chow control	Sari & Zhou (2004)
Alcohol liquid diet: 6.6% (v/v) ethanol + 5-HT_{1A} agonist: Ipsapirone 3 mg/kg	Diet: Pre-gestational & prenatal *(6 weeks before mating-P0)* Agonist: prenatal *(E13-E20)*	N.D.	*Alcohol:* decreased density 5-HT neurons in MRN & DRN (p5); *Agonist:* reversed effects (juvenile)	N.D.	Rat	Sprague Dawley	N.D.	Combination of standard chow & saline injection	Tajuddin & Druse (1999)
Alcohol liquid diet: 6.6% (v/v) ethanol + 5-HT_{1A} agonist: Ipsapirone 1 & 3 mg/kg	Diet: Pre-gestational & prenatal *(6 weeks before mating-P0)* Agonist: prenatal *(E13-E20)*	N.D.	*Alcohol:* decreased density 5-HT neurons MRN & DRN (p5 & p19); *Agonist 3 mg/kg:* reversed effects (juvenile & juvenile)	N.D.	Rat	Sprague Dawley	N.D.	Combination of standard chow & saline injection	Tajuddin & Druse (2001)

Alcohol liquid diet: 5% (w/v) ethanol; 35% ethanol-derived calories	Prenatal *(E8-P0)*	N.D.	Decreased 5-HTT binding sites in hypothalamus (juvenile females); increased sites in amygdala (adolescent & adulthood)	N.D.	Rat	Sprague Dawley	Males & females	Isocaloric-pair-fed liquid control & chow control	Zafar et al. (2000)
Alcohol liquid diet: 3.6% ethanol (v/v); 20% ethanol-derived calories	Prenatal *(E8-E14)*	N.D.	Retarded or no 5-HT neuron migration, differentiation & growth; decreased amount of 5-HT neurons and S100B-cells (E15)	N.D.	Mouse	C57BL/N hsd	N.D.	Ad libitum control diet (standard chow) or pair-fed liquid control diet (isocaloric, alcohol substituted for maltose dextrin)	Zhou et al. (2001)
Isocaloric alcohol liquid diet: 25% ethanol-derived calories	Prenatal *(E7-15)*	N.D.	Fewer 5-HT positive fibres in brain areas including hypothalamus, hippocampus, frontal & parietal cortices; underdevelopment of these brain regions & somatosensory TCA (E15-18)	N.D.	Mouse	C57BL/6	N.D.	Isocaloric-pair-fed liquid control + chow control	Zhou et al. (2005)

3.3. Maternal Stress Affects the 5-HT System, Neurodevelopment, and Behaviour in the Offspring

3.3.2. Prenatal Stress Alters the 5-HT System in the Animal Offspring & 3.3.3. Prenatal Stress-Induced Alterations in Brain Circuits and Behaviour in the Animal Offspring

Restraint stress *(3 times a day 45 min restraint stress)*	Prenatal *(E14-E20)*	Decreased mRNA & protein TPH2 expression in hippocampus & DRN (juvenile males)	N.D.	Increased depressive-like behaviour (juvenile males)	Rat	Sprague Dawley	Males & females	No maternal stress	Dang et al. (2018)
Fluoxetine: 5mg/kg/day and/or restraint stress *(3 times a day (2 times on E21) 45 min restraint stress under bright light)*	Stress: prenatal *(E15-P0)*; SSRI: neonatal *(P0-P21)*	*Fluoxetine & stress:* decreased hippocampus 5-HIAA levels, trend for decreased 5-HT (P21)	*Stress:* increased PFC synaptophysin density (P21)	N.D.	Rat	Sprague Dawley	Males & females	Vehicle (subcutaneously) and/or control stress conditions	Gemmel et al. (2016)

Chronic unpredictable stress: 0–2 stressors per day *(restraint stress under bright light for 1h, cage rotation, overcrowding, food depravation 12h, wet bedding overnight, forced swim)* + Fluoxetine: 10 mg/kg/day	Stress: pre-gestational *(3 weeks-1 day before mating)* SSRI: perinatal *(E10-P21)*	*Stress:* decreased hippocampal 5-HT levels (pre-adolescent females) *SSRI:* reversed effect & increased hippocampal 5-HT levels (pre-adolescent males)	*Stress:* decreased hippocampal neurogenesis & reducing pre-synaptic densities & decreasing immature neurons (males) *SSRI:* increased serum CBG levels & in females increased hippocampal pre-synaptic density (pre- adolescent)	*Stress:* decreased social sibling play *SSRI:* reversed effect	Rat	Sprague Dawley	Males & females	Combination of no maternal stress & saline (wafer biscuit)	Gemmel et al. (2017)
Chronic unpredictable stress: 0–2 stressors per day *(restraint stress under bright light for 1h, cage rotation, overcrowding, food depravation 12h, wet bedding overnight, forced swim)* + Fluoxetine: 10 mg/kg/day	Stress: pre-gestational *(3 weeks-1 day before mating)* SSRI: perinatal *(E10-P21)*	*Stress:* decreased PFC 5-HT levels (pre-adolescent females); *SSRI:* reversed effect (pre-adolescent females)	*Stress:* decreased synaptic markers in mPFC (mainly pre-adolescent & adult males) *SSRI:* decreased GR density of mPFC (adult males)	N.D.	Rat	Sprague Dawley	Males & females	Combination of no maternal stress & saline (wafer biscuit)	Gemmel et al. (2018)
Chronic unpredictable stress: 0–2 stressors per day *(restraint stress under bright light for 1h, cage rotation, overcrowding, food depravation 12h, wet bedding, forced swim)* + Fluoxetine: 10 mg/kg/day	Stress: pre-gestational *(3 weeks-1 day before mating)* SSRI: perinatal *(E10-P21)*	N.D.	*Stress:* decreased hippocampal GR density (females); hippocampal neurogenesis (males); *SSRI:* reversed effects on GR density (adult females); increased hippocampal neurogenesis (adult females); decreased hippocampal synaptic protein (PSD-95) (adult males)	*Stress:* decreased social investigation (adult males); *SSRI:* increased social investigation (adult females) & increased social play (adult males)	Rat	Sprague Dawley	Males & females	Combination of no maternal stress & saline (wafer biscuit)	Gemmel et al. (2019)
Restraint stress *(once a day 2 hours restraint stress)*	Prenatal *(E10-E16)*	Decreased cortex 5-HT metabolism (adult)	Neuroinflammation, decreased oxytocin receptor expression;	Reduced social behaviour and increased CORT after	Mouse	C57Bl6	Males	No maternal stress	Gur et al. (2019)

			alterations in gut microbiome (adult)	social interaction (P60-70)					
Chronic mild psychosocial stress *(daily overcrowding + intramuscular saline injection)*	Prenatal *(E15-P0)*	Decreased hippocampal 5-HT levels (adolescent)	Reduction hippocampal synaptic density (adolescent)	NS spatial learning acquisition test & the probe test (adolescent)	Rat	Wistar	Males & females combined	No maternal stress	Hayashi et al. (1998)
Chronic unpredictable stress: 1 stressor per day at 9.00 *(cage rocking or tilting, swimming cold water, wet bedding, lighting overnight, restraint stress for 12h, food & water deprivation 24h, electric stimulus, elevated temperature)*	Pre-gestational *(23 days-1 day before mating)*	Increased hippocampal & hypothalamic 5-HT levels (E20)	Decreased hippocampal & hypothalamic 5-HTT expression; decreased hippocampal 5-HT_{1A} receptor activity but not in hypothalamic; Increased serum CORT & CRF levels (E20)	N.D.	Rat	Sprague Dawley	Males & females	No maternal stress	Huang et al. (2012)
Chronic unpredictable mild stress *(overcrowding, food deprivation 6h, lighting overnight, cage tilting, foreign object in cage, wet bedding, irregular tones, restraint stress for 2h)* + Fluoxetine: 25 mg/kg/day	Stress: prenatal *(E4-18)* SSRI: perinatal *(E15-P12)*	*Stress:* NS 5-HT levels *SSRI:* decreased whole-brain 5-HT levels (P12)	*Stress:* Decreased fetal frontal cortex BDNF levels (young adulthood)	*Stress:* hyperactivity; NS anxiety-like behaviour *SSRI:* decreased anxiety-like behaviour (young adulthood)	Mouse	C57BL/6	Males	Combination of no maternal stress & normal water (drinking bottle)	Kiryanova et al. (2016)
Restraint stress: strong (6h) or mild (1h)	Prenatal *(E5.5-17.5)*	Enhanced TPH2 immunoreactivities (young adulthood)	*Strong:* increase in 5-HT-positive neurons in DRN (young adulthood)	Increased anxiety-like behaviour; hypo-locomotion (young adulthood)	Mouse	ICR	Males	No maternal stress	Miyagawa et al. (2011)
Maternal restraint stress *(1 time a day 6 hours of restraint stress)* with or without offspring restraint stress *(acute: single restraint stress, 60 min or chronic: 1 time a day 60 min of*	Prenatal *(E5-E17)* Postnatal (offspring) *(P56 or P56-P63)*	*Prenatal + postnatal stress:* decreased TPH in RN (adult)	*Prenatal stress:* decreased Lmx1b in embryonic hindbrain and adult RN	*Prenatal stress:* decreased chronic stress adaptation (adult)	Mouse	ICR	Males	Either maternal stress or offspring restraint stress or neither	Miyagawa et al. (2015)

restraint stress for 7 days)									
Chronic variable stress *(fox odor exposure, 36h constant light, foreign object in cage overnight, restraint stress 5 min., irregular tones overnight, wet bedding overnight, multiple cage changes)*	Prenatal: early *(E1-7)* or middle *(E 8-14)* or late gestation *(E15-21)*	*Early stress:* trend increased mRNA TPH2 expression in DRN (adult males)	*Early stress:* decreased hippocampal 5-HTT levels; NS hypothalamic 5-HTT levels; decreased hippocampal GR; increased amygdala CRF expression & increased serum CORT levels after stress exposure (adulthood)	NS locomotor activity *Early stress:* increased depressive-like behaviour (adulthood)	Mouse	C57BL/6: 129	Males & females	No maternal stress	Mueller & Bale (2008)
Chronic mild psychosocial stress *(daily overcrowding + subcutaneous saline injection)*	Prenatal *(E16-P0)*	Increased TRP & 5-HT levels in fetal total brain (E20) & neonatal cerebral cortex (males P0-P10 & females P0)	N.D.	N.D.	Rat	Sprague Dawley	Males & females	No maternal stress	Peters (1990)
Restraint stress *(3 times a day 45 min restraint stress under bright light, 2 times on E21)* + Fluoxetine: 5 mg/kg/day	Stress: prenatal *(E15-21)*; SSRI: neonatal *(P0-21)*	N.D	***Stress:* decreased hippocampal cell proliferation & neurogenesis *SSRI:* reversed effect (late adolescent)**	*Stress:* increased anxiety-like but decreased depressive-like behaviour; NS locomotor activity *SSRI:* reversed effect on depressive-like behaviour (adolescent)	Rat	Sprague Dawley	Males & females	Combination of no maternal stress & vehicle (50% propylenediol in saline) treatment (osmotic pump)	Rayen et al. (2011)
Restraint stress *(3 times a day 30 min restraint stress)* + Fluoxetine: 8 mg/kg/day	Stress: prenatal *(E5-19)*; SSRI: perinatal *(E10-P20)*	N.D.	*Stress*: increased HPA-axis reactivity *SSRI:* reversed effect (adulthood)	*Stress:* increased anxiety-like & depressive-like behaviour; NS locomotor activity *SSRI:* reversed effect (adulthood)	Mouse	NMRI	Males	Combination of no maternal stress & water treatment (drinking bottle)	Salari et al. (2016)
Chronic unpredictable stress: 3 stressors per week *(restraint*	Prenatal *(E3-E20)*	Decreased hippocampal 5-HT levels (adult)	Decreased stria terminalis volume; increased CORT and HPA-response to acute stress (adult);	Increased anxiety-like behaviours & depressive-like behaviours; NS	Rat	Wistar	Males & females	No maternal stress	Soares-Cunha et al. (2018)

stress for 4 hours, stroboscopic lights for 4 hours or exposure to noise (80dB for 4 hours))			Decreased neuron count in bed nucleus of stria terminalis (adult females) Decreased volume and neuron count in ventral hippocampus; increased volume in dorsal hippocampus (adult males)	locomotor activity & impulsity (adult)					
Maternal restraint stress *(3 times a day 45 minutes of restraint stress under bright light)* with or without offspring chronic unpredictable mild stress: 2 stressors per day *(3 hours: housing in mouse cage, 45° cage tilt, empty cage, wet bedding, stroboscopic light)*	Prenatal *(E14-E21)* Postnatal (offspring) *(P77-P98)*	*Prenatal stress:* Alterations in amount of THP2 in dentate gyrus & hippocampus (mostly males); decreased DRN 5-HT relative to control; Increased prelimbic cortex & infralimbic cortex 5-HT (adult males)	*Prenatal stress:* increased HPA-axis reactivity (adult males)	*Prenatal stress:* increased anxiety-like behaviour (adult)*:* increased depressive-like behaviour; *Chronic mild stress:* normalized effects of prenatal stress on HPA-axis reactivity & anxiety-like behaviour in elevated zero maze	Rat	Sprague Dawley	Males & females	Either maternal stress or offspring stress or neither	Van den Hove et al. (2014)
Chronic unpredictable stress: 1 stressor a day *(odour exposure, lighting overnight, social isolation, irregular tone overnight, cage tilting, wet bedding overnight, intraperitoneal injection)* + citalopram: 260 mg/L	Prenatal *(stress & SSRI: E8-E17)*	*Stress:* increased forebrain & frontal cortex 5-HT levels *SSRI:* reversed effect (E17)	*Stress:* increased the numbers of deep-layer neurons in specific cortical regions *SSRI:* reversed effect via increase of overall cell numbers without changing proportions of layer-specific neurons (E17)	N.D.	Mouse	CD-1	Males & females combined	Combination of no maternal stress & normal water (drinking bottle)	Velasquez et al. (2019)
Maternal social defeat stress	Pre-gestational *(3 weeks stress, then 1 week rest, then breeding)*	Decreased hippocampus, hypothalamus & PFC 5-HT (adult)	Increased CORT & ACTH, decreased PFC and hippocampus BDNF and pCREB; increased	Increased anxiety, increased depressive-like behaviour; reduced memory retention; hypo-locomotion (adult)	Rat	Wistar	Males	No maternal stress	Wei et al. (2018)

			hippocampus & PFC 5-HTT (adult)						
Stressors: restraint, forced swim, elevated platform + Citalopram: 10 mg/kg/day	Stress: prenatal *(E13-21)* SSRI: perinatal *(E7-P21)*	N.D.	*Stress:* reduction 5-HT$_{1A}$ receptor in PFC (adult males)	*Stress:* increased anxiety-like & depressive-like behaviour *SSRI*: increased anxiety-like (males only) & depressive-like behaviour (adulthood)	Rat	Wistar	Males & females	Healthy animals (drinking bottle)	Zohar et al. (2016)

3.4. Maternal Intake of 5-HT-ergic Medication Alters 5-HT Levels in the Offspring and Affects their Neurodevelopment and Behaviour

3.4.3. Maternal SSRI Intake Affects the Animal Offspring's 5-HT Signalling and Behaviour & 3.4.4. Maternal SSRI Intake Affects the Animal Offspring's brain circuitry development

Prenatal SSRI intake

Fluoxetine: 8 or12 mg/kg/day	Prenatal *(E6-E20)*	N.D.	N.D.	*Both doses:* initial transient delay in motor development (juvenile); NS anxiety-like behaviour (adolescent)	Rat	Wistar	Males & females	Distilled water (orally)	Bairy et al. (2007)
SSRI: Escitalopram oxalate: 12.2 mg/kg/day and/or *Stress:* Chronic unpredictable mild stress *(restraint, cage tilt, damp bedding, cage changes, noise, and overnight illumination)*	Prenatal *(SSRI: at least 3 days before E0-E21; Stress: E9-E20)*	N.D.	*SSRI:* increased amygdala 5-HT$_{1A}$ receptor *Stress:* altered amygdala gene expression (GABAergic function-related) (adolescent, not adult)	*SSRI:* reduced social interaction (adolescent, not adult) *Stress:* increased anxiety-like behaviour, decreased performance object recognition tests (adult)	Rat	Sprague Dawley	Females	No maternal stress, saline (osmotic pump)	Ehrlich et al. (2015)
Fluoxetine: 20 mg/kg/day	Prenatal *(E14.5-E18.5）*	N.D.	Increased migratory speed of inhibitory cortical interneurons (E17.5); affect transcriptional programmes	N.D.	Mouse	C57BL/6	N.D.	Placebo pellet placed subcutaneously	Frazer et al. (2015)

			regulating neuronal migration (E18.5)						
Fluoxetine: 12 mg/kg/day	Prenatal *(E11-P0)*	N.D.	Decreased Npas4 in hippocampus & PFC (adulthood)	N.D.	Rat	SERT WT Slc6a41Hubr	N.D.	Methylcellulose (orally)	Guidotti et al., 2012
Citalopram: 20 mg/kg/day	Prenatal *(last 7 days of gestation)*	N.D.	In the mPFC: downregulation NMDAR1 & CaMKIIα and increased parvalbumin-positive cells; increase cFOS in mPFC & striatum; increased mPFC-striatal synchronization; aberrant PFC oscillations (adulthood)	N.D.	Mouse	C57BL/6	Males & females	Saline (intraperitoneal)	Jiang et al. (2019)
Citalopram: 20 mg/kg/day with 1% sucrose	Prenatal *(E8-17)*	N.D.	Decreased P11 protein expression in thalamus; decreased neurogenesis (E17)	N.D.	Mouse	CD-1	N.D.	Regular water with 1% sucrose (drinking bottle)	King et al. (2017)
Fluoxetine: 12 mg/kg/day	Prenatal *(E11-P0)*	N.D.	N.D.	Decreased social play (adolescent); increased anxiety-like behaviour; NS anhedonia (adulthood)	Rat	Wistar	Males	Methylcellulose (orally)	Olivier et al. (2011)
Citalopram: 20 mg/kg/day (dams); 10 mg/kg/day (pups P1-7); 5 or 20 mg/kg/day (pups p8-21)	Prenatal *(E11-19)* or Neonatal *(P1-7 or P8-21)*	N.D.	*All citalopram exposed offspring:* changed oligodendrocytes morphology in the corpus callosum & altered axon myelination in the corpus callosum (mainly due to neonatal treatment); reduced connectivity between the primary	*Neonatal p8-21 20 mg/kg:* decreased juvenile social play (males) & neophobia (juvenile & adolescent)	Rat	N.D.	Males & females	Saline	Simpson et al. (2011)

			somatosensory cortices across the hemispheres (adulthood)						
Fluoxetine: 0.6 mg/kg/day	Prenatal *(E8-18)*	N.D.	Reduced complexity of the dendrites of cortical layer 2/3 pyramidal neurons (juvenile & (young) adulthood)	Increased anxiety-like behaviour (adulthood)	Mouse	C57BL/6J	N.D.	Saline (intraperitoneally)	Smit-Rigter et al. (2012)
Fluoxetine: 10 mg/kg/day	Prenatal *(E1-P0)*	N.D.	N.D.	Delayed emergence of maternal behaviour (adult females)	Mouse	CD-1	Males & females	Saline (subcutaneously)	Svirsky et al. (2016)
Fluoxetine: 0.6 mg/kg/day	Prenatal *(E4-E19)*	N.D.	Increase in 5-HT_{2A} receptor expression & trend for increase in 5-HT_{1A} receptor expression in prelimbic area of PFC; increased miniature inhibitory synaptic currents in pyramidal layer 5 of the PFC	Decreased working memory and social recognition performance (adult)	Mouse	C57BL/6J	Males	Saline (intraperitoneally)	Yu et al. (2019)
SSRI: Fluoxetine: 5 mg/kg/day and/or *Stress:* Chronic unpredictable stress, 2 stressors per day *(restraint stress: 15 min, cat odour exposure: 60 min, fox odour exposure: 5 min, forced swim: 1 min, open field: 6 min, maze exploration: 5 min, footshocks: 15x500 ms, intraperitoneal saline, food deprivation: 14 h)*	Pre-gestational *(Stress: P45-P51; SSRI: P52-P59; Breeding performed on: P66)*	N.D.	*SSRI & stress:* affected expression of mRNA editing enzymes in PFC & amygdala differentially between groups *Stress:* altered 5-HT_{2C} receptor editing and GLUT receptor editing in PFC & amygdala *SSRI:* decreased editing at 5-HT_{2C} receptor in amygdala but enhanced 5-HT_{2C} receptor editing enzyme expression in PFC; reversed effect	*SSRI:* enhanced social preference (males only, adult)	Rat	Sprague Dawley	Males & females	No maternal stress & saline (intraperitoneally)	Zaidan et al. (2018)

			of stress on 5-HT_{2C} receptor in PFC (P0)						
Neonatal SSRI intake									
Citalopram: 10 or 20 mg/kg	Neonatal *(once between P2-5)*	N.D.	Suppressed amplitude & prolonged delay of sensory-evoked potentials, reduced power & frequency of early gamma oscillations, suppressed sensory evoked & spontaneous neuronal firing in the barrel cortex (pup)	N.D.	Rat	Wistar	Males & females combined	Unhandled control & saline control (intraperitoneally)	Akhmetshina et al. (2016)
Escitalopram or Fluoxetine: 10 mg/kg/day	Neonatal *(P5-P21)*	*Escitalopram:* decreased extracellular hippocampal 5-HT *Fluoxetine:* NS (adulthood)	*Escitalopram:* decreased 5-HTT binding in MRN & trend MRN *Fluoxetine:* NS (adulthood)	*Escitalopram:* decreased anxiety-like behaviour; NS depressive-like behaviour (adolescent & adulthood) *Fluoxetine:* increased anxiety-like behaviour & reduced exploration; NS depressive-like behaviour (adulthood)	Mouse	5-HTT WT CD-1 129SvEv	Males & females	Unhandled control & saline control (subcutaneously)	Altieri et al. (2015)
Fluoxetine: 10 mg/kg/day	Neonatal *(P4-P21)*	N.D.	N.D.	Increase in anxiety-like behaviour in 5-HTT WT/HET mice (young adulthood)	Mouse	5-HTT KO/HET/WT	Males & females combined	Saline (intraperitoneally)	Ansorge et al. (2004)
Fluoxetine: 5 or 10 mg/kg/day; desipramine: 5 or 10 mg/kg/day; citalopram: 5 or 10 mg/kg/day; clomipramine: 5 or 20 mg/kg/day	Neonatal *(P4-P21)*	N.D.	N.D.	*All except desipramine:* increased anxiety-like behaviour (adulthood)	Mouse	5-HTT HET/WT 129S6/SvEv	Males & females combined	Saline (intraperitoneally)	Ansorge et al. (2008)

Fluoxetine: 10 mg/kg/day + sesame oil: 1 ml/kg/day	Neonatal *(P2-P23)*	N.D.	Increased HPA-axis CORT release (adult); increased density of doublecortin-expressing neurons in dorsal hippocampus (adult males) Decreased density of doublecortin-expressing neurons in dorsal hippocampus (adult females)	Increased anxiety-like behaviour (adult males) Increased swimming time in forced-swim test (adult)	Rat	Sprague Dawley	Males & females combined	Saline (intraperitoneally) + sesame oil (subcutaneously)	Gobinath et al. (2016)
Citalopram: 5 or 10 or 20 mg/kg/day	Neonatal *(P8-P21)*	N.D.	N.D.	NS difference in anxiety-like behaviour (adulthood)	Rat	Long Evans	Males	Saline (subcutaneously)	Harris et al. (2012)
Citalopram: 20 mg/kg/day	Neonatal *(P8-P21)*	N.D.	N.D.	Decreased social behaviour (juvenile); increased freezing after tone (adolescent), increased stereotypic behaviour & neophobia (adolescence/adulthood)	Rat	Long Evans	Males & females	Saline (subcutaneously)	Khatri et al. (2014)
Fluoxetine: 20 mg/kg/day	Neonatal *(P0-P4)*	NS TPH2, 5-HT or 5-HIAA in, amongst others, DRN & MRN (adulthood)	Increased dendritic complexity & length and reduced dendritic spine count in pyramidal layer II & III of the mPFC; decreased dendritic spine density of pyramidal neurons in basolateral amygdala (adulthood)	Decreased locomotor activity, increased depression-like behaviour, decreased PPI (adulthood)	Rat	Wistar	Males	Saline (subcutaneously)	Ko et al. (2014)
Fluoxetine: 10 mg/kg/day	Neonatal *(P1-6)*	N.D.	Reduced branch tips of TCAs to somatosensory cortex; reduced dendritic span & complexity with fewer branches,	Blunt thermal & tactile perceptions; decrease in exploration (adolescence)	Rat	Wistar	Males & females combined	Saline (subcutaneously)	Lee (2009)

			shorter dendritic length, smaller dendritic field (juvenile)						
Restraint stress *(3 times a day 45 min restraint stress under bright light, 2 times on E21)* + Fluoxetine: 5 mg/kg/day	Stress: prenatal *(E15-21)* SSRI: neonatal *(P1-21)*	N.D.	*SSRI:* Decreased serum CORT levels & free CORT index; decreased hippocampal GR & GRIP1 density (adolescent males)	N.D.	Rat	Sprague Dawley	Males & females	Combination of no maternal stress and vehicle (50% propylenediol in saline) treatment (osmotic pump)	Pawluski et al. (2012b)
Escitalopram: 10mg/kg/day	Neonatal *(P5-P19)*	N.D.	Increased 5-HT_{1A} receptor function in raphe 5-HT-ergic neurons; larger increase in CORT levels directly after stressor (adulthood)	Increased depressive-like behaviour including anhedonia (adulthood); NS anxiety-like behaviour	Mouse	Swiss CD-1	Females	Saline (subcutaneous)	Popa et al. (2008)
Fluoxetine: 10 mg/kg/day	Neonatal *(P2-11)*	N.D.	Dendritic hypotrophy pyramidal neurons in mPFC; decreased excitability of infralimbic pyramidal neurons; increased excitability of prelimbic pyramidal neurons (adulthood)	Increased anxiety- & depressive-like behaviour; impaired fear extinction (adulthood)	Mouse	129S6/Sv EvTac	Males & females	Saline (intraperitoneal)	Rebello et al. (2014)
Citalopram: 20 mg/kg/day or Fluoxetine: 10 mg/kg/day	Neonatal *(P8-P21)*	N.D.	N.D.	Increased freezing after tone (adolescent), decreased novel object interaction, decreased social interaction; decreased male sexual behaviour (adulthood)	Rat	Long Evans	Males & females	Saline (subcutaneously)	Rodriguez-Porcel et al. (2011)
Paroxetine: 10 mg/kg/day	Neonatal *(P1-8)*	N.D.	Disrupted organization of TCAs: reduced barrel size & enlarged septa (pre-adolescent)	N.D.	Rat	Sprague Dawley	N.D.	Saline (subcutaneously)	Xu et al. (2004)
Fluoxetine: 10 mg/kg/day	Neonatal *(P2-7)*	N.D.	N.D.	Increased risk of ASD: decreased	Rat	NIH Norway;	Males & females	No manipulation & sucrose	Zimmerberg &

				social play & interaction; NS anxiety-like behaviour (juvenile & adulthood)		low & high line			Germeyan (2015)
Perinatal SSRI intake									
Fluoxetine: 15 mg/kg/day	Perinatal *(E1-P14)*	N.D.	Reduced MAOA mRNA expression (juvenile)	Disrupt sociability; reduce preference for social novelty (juvenile until young adulthood)	Mouse	C57BL/6	Females	Normal water (drinking bottle)	Bond et al. (2019)
Fluoxetine: 10 mg/kg/day	Perinatal *(E1-P21)*	N.D.	N.D.	Increased social interaction when studying in a semi-natural environment; altered stress coping behaviour (adulthood)	Rat	Wistar	Males & females	Methylcellulose (orally)	Houwing et al. (2019)
Fluoxetine: 12 mg/kg/day	Perinatal *(E11-P7)*	N.D.	N.D.	Delayed motor & reflex development (juvenile & adolescent)	Rat	Wistar	Males	Methylcellulose (orally)	Kroeze et al. (2016)
Fluoxetine: 25 mg/kg/day	Perinatal *(E15-P12)*	NS whole-brain 5-HT levels (P1); reduced whole-brain 5-HT levels (P12)	N.D.	Improved spatial memory, decreased anxiety-like behaviour (adulthood)	Mouse	C57BL/6	Males	N.D.	Kiryanova & Dyck (2014)
Fluoxetine: 7.5 mg/kg/day	Perinatal *(E1-P21)*	N.D.	N.D.	Decreased impulsivity (adult males); increased depressive-like behaviour (adolescent & adult females)	Mouse	Swiss	Males & females	Tap water (orally)	Lisboa et al. (2007)
Fluoxetine: 16 mg/kg/day + 1% saccharin	Perinatal *(E0-P14)* or long prenatal *(E0-P0)* or short prenatal *(E0-E16);*	N.D.	N.D.	*Perinatal & long prenatal:* Decreased early communicative behaviour *Long prenatal:* Decreased social behaviours (adulthood) *Perinatal:* increased repetitive behaviours;	Mouse	C57BL/6J	Males & females	1% saccharin water (orally)	Maloney et al. (2018)

				tactile hypersensitivity (adulthood)					
Fluoxetine: 25 mg/kg/day	Perinatal *(E15-P12)*	N.D.	N.D.	Decreased anxiety & depressive-like behaviour; NS locomotor activity, PPI, startle response, multiple memory tests (adulthood)	Mouse	C57BL/6	Males & females	Normal water (drinking bottle)	McAllister et al. (2012)
Sertraline: 5 mg/kg/day (dams); 1.5 mg/kg/day (pups)	Perinatal *(E1-P14)*	Increase in cerebral cortex TPH2 mRNA (adulthood)	Increased 5-$HT_{1A/2A/2C}$ receptors & 5-HTT mRNA expression in cerebral cortex (adulthood)	NS social interaction, spatial learning, explorative behaviour (adulthood)	Mouse	C57BL/6	Males & females combined	Saline (intraperitoneal)	Meyer et al. (2018)
Fluoxetine: 5mg/kg/day	Perinatal *(E1-P21)*	N.D.	Increased DNA methylation in hippocampus, reduced plasma CORT after restraint stress (adulthood)	Decreased social interaction time; NS difference in elevated plus maze test (adulthood)	Rat	Wistar	Male	Water (orally)	Silva et al. (2018)
Citalopram: 20 mg/kg/day	Perinatal *(E6-P20)*	N.D.	N.D.	Induced ASD-like behaviour: decreased PPI; increased anxiety-like & depressive-like behaviour (young adulthood)	Rat	Sprague Dawley	Males & females	Saline (subcutaneously)	Sprowles et al. (2016)
Fluoxetine: 11.3 ±0.1 mg/kg/day & methylmercury: 0.59 mg/kg/day	Perinatal *(E7-P7)*	N.D.	Decreased perineuronal net formation; NS number parvalbumin neurons in hippocampus & amygdala	N.D.	Mouse	C57/BL6J	Males & females combined	Tap water (drinking bottle)	Umemori et al. (2015)

3.5. Maternal immune activation affects the tryptophan pathway and neurodevelopment of offspring

3.5.1. Activation of the fetal immune system influences the animal offspring

Cytokine exposure: IL-6 (100 U/ng); IL-	Dissection brain tissue at E14	N.D.	Decreased survival rostral RN 5-HT neurons	N.D.	Rat	Sprague Dawley	N.D.	Serum-free medium without cytokines	Jarskog et al. (1997)

1β (50 U/ng); TNF-α (60 U/ng)									
**3.5.2. Changes in Placenta-Derived 5-HT Levels Influence the Animal Offspring**									
Poly(I:C): 2 mg/kg + TPH1 inhibitor: para-cholorophen-ylalanine 300 mg/kg	E12	*Poly(I:C):* increased forebrain 5-HT & kunurenine, NS hindbrain (E14) *Inhibitor:* reversed effects (E14)	*Poly(I:C):* decreased 5-HT axon density in rostral two-thirds of the forebrain (E14) *Inhibitor*: reversed effects (E14)	N.D.	Mouse	CD-1	Males & females	Saline injection	Goeden et al. (2016)
Lipopolysaccharide injection: 100 µg/kg, Escherichia coli endotoxin	25 µg/kg on E15, 25 µg/kg on E16, 50 µg/Kg on E17	NS cortical 5-HT levels (E18) but decreased 5-HT levels (adolescent/adult)	Decreased TPH1 mRNA expression (E18); increased TPH1 but decreased TPH2 and 5-HTT mRNA expression, decreased amount of TPH2 expressing cells in RN (adolescent/adult)	Increased anxiety-like behaviours (5, 6 & 9 weeks old)	Mouse	C57BL/6	Females	Phosphate-buffered saline injections (subcutaneously)	Hsueh et al. (2017)
20 mg/kg of Escherichia coli endotoxin	E28	Decreased 5-HT levels in (frontal & parietal) cortex & hippocampus (p1)	Decreased 5-HT-immunoreactive fibres in somatosensory cortex; decreased 5-HTT mRNA expression in parietal sensory cortex; loss of thalamic neurons & TCA; NS raphe 5-HT-ergic cell bodies (p1)	N.D.	Rabbit	New Zealand	N.D.	No treatment control & saline injection control	Kannan et al. (2011)
Poly(I:C): 10 mg/kg	E9	Decreased hippocampal 5-HT levels, NS striatum (young-adult)	Increased number of 5-HT-ergic neurons in rostral RN (E15)	N.D.	Rat	Wistar	N.D.	PBS injection	Ohkawara et al. (2015)
Lipopolysaccharide injection: ~8,000 EU of Escherichia coli endotoxin	E28	Decreased fetal hippocampal & thalamic 5-HIAA; increased IDO &	N.D.	N.D.	Rabbit	New Zealand	N.D.	No treatment except intravenous fluids	Williams et al. (2017)

		kynurenine & quinolinic- & kynurenine- acid periventricular white matter region (E29)							

3.5.3. Maternal Immune System Activation Influences Brain Circuits and Behaviour in the Animal Offspring

Influenza administration	E18	Reduced 5-HT (P14 & P35), reduced 5-HIAA (P14)	Altered gene expression & protein levels in frontal, hippocampal and cerebellar cortices; brain atrophy & thinned white matter of corpus callosum (P35)	N.D.	Mouse	C57BL/6J	Males	Vehicle injection	Fatemi et al. (2009)
Poly(I:C): 4mg/kg	E15	Reduced 5-HIAA in mPFC & hippocampus, reduced 5-HT in caudate-putamen & globus pallidus, increased 5-HT in ventral tegmental area (adolescent/adult)	N.D.	Reduced PPI (adult but not adolescent)	Rat	Wistar	Males	Saline injection	Hadar et al. (2015)
Influenza administration: 75 pfu or 300 pfu	E9	Dose-dependent decrease in 5-HT & increase in 5-HIAA (adult)	Reduced oxytocin (adult) *300 pfu:* increased microglia density in brainstem nuclei (adult males)	Dose-dependent reduction in social behaviours and increased violent behaviours (adult) *75 pfu:* decreased locomotor activity and increased anxiety-like behaviour (adult females) *300 pfu:* increased locomotor activity and decreased anxiety-like behaviour (adult males)	Mouse	BALB/c	Males & females	Saline administration (intranasally)	Miller et al. (2013)

Poly(I:C): 20 mg/kg	E12.5	N.D.	Increased hippocampal 5-HTT protein expression (adulthood)	Increased depressive-like behaviour; NS anxiety-like behaviour (adulthood)	Mouse	C57BL/6 N	Females	Saline injection	Reisinger et al. (2016)
Influenza A/NWS/33 (H1N1) administration	E16	Decreased cerebellum 5-HT (P14, NS P0 & P56)	N.D.	N.D.	Mouse	C57BL/6J	Males	Saline administration (intranasally)	Winter et al. (2008)
Poly(I:C): 5 mg/kg	E9	Decreased 5-HT in nucleus accumbens, decreased 5-HT & 5-HIAA in lateral globus pallidus & hippocampus	NS GABA & glutamate changes	N.D.	Mouse	C57BL/6J	Males	Saline injection (i.v.)	Winter et al. (2009)

5-HT: serotonin; 5-HTT: serotonin transporter; 5-HIAA: 5-hydroxyindole amino acid; CBG: corticosteroid binding globulin; CORT: corticosterone; CRF: Corticotropin-Releasing Factor; CSF: cerebrospinal fluid; DRN: dorsal raphe nuclei; E: embryonic day; GR: glucocorticoid receptor; HET: heterozygotic; HPA: hypothalamus-pituitary-adrenal; IDO: indoleamine 2,3-dioxygenase; KI: knockin; KO: knockout; MAOA: monoamine oxidase A; mPFC: medial prefrontal cortex; MRN: median raphe nuclei; N.D.: not determined; NS: not significant; P: postnatal day; PFC: prefrontal cortex; PM: postnatal month; PPI: pre-pulse inhibition; Poly(I:C): polyriboinosinic-polyribocytidylic acid; PW: postnatal week; RN: raphe nuclei; SSRI: selective serotonin reuptake inhibitor; TCA: thalamocortical afferents; TPH: tryptophan hydroxylase; TRP: tryptophan; WT: wildtype.
